# Quality of life among residents of informal urban settlements during the COVID-19 pandemic in Salvador, Brazil

**DOI:** 10.1136/bmjph-2023-000572

**Published:** 2024-05-27

**Authors:** Hammed Mogaji, Nivison Nery Jr, Hernan D Argibay, Jaqueline S Cruz, Ianei O Carneiro, Ricardo Lustosa, Albert I Ko, Federico Costa, Mike Begon, Hussein Khalil

**Affiliations:** 1 Gonçalo Moniz Institute, Oswaldo Cruz Foundation, Brazilian Ministry of Health, Salvador, Bahia, Brazil; 2 Institute of Collective Health, Universidade Federal da Bahia, UFBA, Salvador, Brazil; 3 Department of Epidemiology of Microbial Diseases, Yale School of Public Health, New Haven, Connecticut, USA; 4 Faculdade de Medicina da Bahia, Universidade Federal da Bahia, Salvador, Brazil; 5 Department of Evolution, Ecology and Behaviour, University of Liverpool, Liverpool, UK; 6 Swedish University of Agricultural Sciences, Umeå, Sweden

**Keywords:** COVID-19, Community Health, Public Health

## Abstract

**Background:**

Disadvantaged populations, especially those living in rural and informal settlements, constitute the most affected during the COVID-19 pandemic. However, there is limited information on the health-related quality of life (HRQOL) and indirect consequences of non-pharmaceutical interventions implemented during the pandemic.

**Methods:**

We leveraged on an ongoing prospective open-cohort survey and performed a cross-sectional analysis of data collected between November 2021 and July 2022 among 793 residents above age 5 in a large slum community in the city of Salvador, Brazil. We describe the HRQOL of residents, explored participants’ perception about the pandemic, and the relationship between sociodemographic, economic and employment data on physical and mental health scores using both χ^2^ statistics and separate mixed-effects regression models.

**Results:**

Most participants were female (58.9%), aged 18–45 years (45%), with nearly half (49.7%) employed before pandemic, of whom 38.8% lost jobs during the pandemic. Food insecurity was 69.6%, and only 27.1% received government aid. Those retaining employment during the pandemic had better physical (β: 4.02, 95% CI 1.34 to 6.69, p<0.001) and mental (β: 3.08, 95% CI 0.60 to 5.56, p<0.001) health. Females had lower physical health scores than males (β: −2.44, 95% CI −3.94 to −0.94, p=0.002). Older participants had lower physical health scores (β: −9.11, 95% CI −12.14 to −6.07, p<0.001), but higher schooling improved physical health (p<0.001). Females and older adults faced more COVID-impacted challenges (p<0.001) related to health, education, family, social relationships, work, finances and employment.

**Conclusion:**

We found lower HRQOL among adults, females, the unemployed and those with lower school attainment. In addition, women and individuals in older age groups reported experiencing COVID-impacted mental challenges more frequently than others. These findings highlight the need to prioritise creation of economic opportunities and expansion of existing assistance programmes for marginalised populations residing in these slums.

**WHAT IS ALREADY KNOWN ON THIS TOPIC**

**WHAT THIS STUDY ADDS**

- The study reveals a worrisome unemployment rate, food insecurity and coverage of COVID-19 emergency fund during the pandemic.
- Retaining employment during the pandemic significantly improved both physical and mental health of participants.
- Females and older adults faced more COVID-impacted challenges in various aspects of life.

**HOW THIS STUDY MIGHT AFFECT RESEARCH, PRACTICE OR POLICY**

- The findings of this study will likely influence policymakers and practitioners to provide employment opportunities, and interventions focused on vulnerable female and older populations.

## Introduction

 The novel coronavirus disease, COVID-19, is caused by the SARS-CoV-2 and remained a global pandemic from 2020 until 2023. Over 769 million cases and 6.9 million deaths have been reported, with the USA, India and Brazil topping the list of the most affected countries.^1 2^ Brazil has recorded over 37 million cases and 704 659 deaths due to COVID-19 (4.8% of the worldwide total) and has been the most affected country in Latin America.^1 2^ The majority of those affected by the pandemic are residents of urban slums or informal settlements,^3^ where there is a lack of basic infrastructure such as water, toilets, sewers, drainage, waste collection and adequate housing.^4^,^9^ Beyond COVID-19, lack of access to urban services and inadequate infrastructure can increase the vulnerability to other infectious diseases, thus indicating the need for a healthier socio-spatial environment to reduce transmission and increase resilience to health or environmental hazards.^10^

Until vaccines were developed and introduced in the latter stages of 2020,^11^ the SARS-CoV-2 virus was primarily countered through the blanket implementation of non-pharmaceutical interventions (NPIs). These NPIs included measures such as travel restrictions, event cancellations, curfews and lockdowns.^12^ In workplaces, there was imposition of lockdowns on businesses, downsizing and recommendations to work from home.^13^ In addition, other hygienic measures such as social distancing, regular handwashing and facemask usage were instituted. These NPIs contributed significantly to the decline in transmission,^14^ but there were also unintended socioeconomic consequences on income,^15^ mental health^16^ and physical activities.^17^

The COVID-19 pandemic has starkly magnified the existing social inequalities experienced by vulnerable communities, particularly those comprising informal workers who rely on daily wages for their livelihoods.^18 19^ Within the realm of global health, social justice and equity take centre stage, emphasising the crucial need to prioritise the concerns of marginalised populations already burdened by an unequal distribution of risk factors and disease. Unfortunately, the response to the pandemic has revealed the shortcomings of a one-size-fits-all approach, perpetuating inequity, and potentially deepening disparities in the future. Notably, the economic repercussions of widespread lockdowns have disproportionately impacted approximately 2 billion people who depend on the informal economy for their survival, with over 90% of them residing in low-income and low to middle-income countries.^20^

In Brazil, recent economic struggles led to 36% of the population becoming unable to afford sufficient food daily. This problem is particularly pronounced in the northeast region.^21^ Additionally, the unemployment rate has increased by 10 percentage points during the pandemic, reaching 36%, and vulnerable slum populations have experienced a 9.4% reduction in average individual income.^21^ These trends may exacerbate the burden of the pandemic on the general well-being of individuals residing in urban slums. A significant area of worry is how the NPIs have affected the socioeconomic standing of slum communities, which has not been thoroughly researched. Additionally, there is a lack of studies that examine the health-related quality of life (HRQOL), encompassing both physical and mental health, of these populations during the pandemic. This information is crucial to aid government recommendations and future pandemic preparedness. We therefore describe the HRQOL of residents in a major slum community in Salvador, Brazil, during the pandemic. In addition, we explored their perception of the pandemic on mental and overall well-being, and investigated the relationship between sociodemographic, economic and employment data on physical and mental health outcomes. The findings will be useful for the establishment of health policies and strategies to mitigate the effects of pandemics in the future.

## Materials and methods

### Study settings and population

This study leveraged an ongoing open-cohort epidemiological project in the community of Marechal Rondon, an urban slum in the city of Salvador, Bahia, north-eastern Brazil. This area is characterised by poor social and environmental conditions, including inadequate infrastructure and sanitation, as well as a high concentration of families residing in vulnerable areas at risk.^22^ The community has a clear altitudinal gradient with open sewers, unpaved roads and households without easy access to the main roads.^4^ The selection of the study area followed a systematic approach, initially focusing on an area previously studied for exposure to leptospirosis and other arboviruses.^4^ This area was further delimited to a range of 0.07–0.09 km^2^, taking into account residents’ vulnerability, particularly those in close proximity to an open sewer that traverses the community. A recent serological survey within this defined sampling area suggested that a sample size of 338 would be adequate, considering a 12% seroprevalence for leptospirosis, a 95% CI and an assumed non-response rate of 10%.^4^ However, given the comprehensive nature of our investigation, we made efforts to include all eligible residents by visiting every residential building within the study sites. Our census identified a total of 1596 residents, with only 908 (57%) consenting to participate in the study. From this population, only 793 individuals had complete data for the study indicators considered in our analysis. The seropositivity for SARS-CoV-2 among population above age 5 was estimated at 91% during the period of this study.

### Study procedure

We performed a cross-sectional analysis of data collected between November 2021 and July 2022 among 793 residents above age 5 in a large slum community in the city of Salvador, Brazil. In brief, the project recruited individuals who reside in the community (defined as sleeping three nights or more per week), and were aged 5 years or more. Biannual serological surveys involving collection of biological samples (blood, nasal swabs and saliva) and collections of epidemiological data using standardised questionnaires were conducted. A trained field research team comprising interviewers, local guides and phlebotomists performed door-to-door visits to explain the objectives of the research and recruitment of participants. The process of recruitment involved obtaining written informed consent or assent forms prior to data collection using standardised questionnaires. Participants below age 18 provided written informed assent and their legal guardians provided written informed consent. Trained phlebotomists then collected blood samples which were labelled using a unique participant code. These samples were transported to the laboratory in iceboxes within a 4-hour timeframe from collection. Subsequent analysis involved the use of immunoassay kits to identify antibodies, specifically immunoglobulin G, for SARS-CoV-2, arboviruses (dengue and chikungunya) and leptospirosis. The epidemiological findings and associated risk factors resulting from these analyses fall beyond the purview of the current study and will be presented separately.

### Study instrument and data collection

This study used a close-ended electronic-based questionnaire to collect epidemiological data from consenting residents. The questionnaire was designed, administered and managed using the Research Electronic Data Capture (REDCap) software.^23 24^ In brief, the questionnaire had been validated and used for over a decade by our wider team in another neighbouring community within Salvador.^25 26^ The questionnaire consists of two parts: the household survey which documents information about the household and is administered to the head of household, and the individual survey which documents the sociodemographic, economic, knowledge and perceptions of everyone in the household, and, here, the perceived effect of the pandemic on mental health, and a variety of aspects of their general well-being including family and social relationships, education, finances, employment, hunger, violence, security and access to healthcare. Study indicators include demographic, socioeconomic and environmental factors (gender, age, educational status, income, race/ethnicity, access to water and sanitation facilities, type of employment, means of transportation to work and access to food supply).

Due to the pandemic, modifications were made to our questionnaire to incorporate new questions that explore employment opportunities and HRQOL following the pandemic. To assess HRQOL, we incorporated the 12-Item Short Form Survey (SF-12) health questionnaire targeted at participants aged 12 and above.^27 28^ The questionnaire has two components: the physical health component that describes concerns about physical functioning, difficulties and pain associated with mobility, and the mental health component that describes concerns about depression, anxiety, accomplishments, emotional role and participation in social activities. The questionnaire employs a scoring system to evaluate the physical and mental health of participants.^27 28^ A Portuguese-translated version of the instrument, which has been validated in an earlier study, was then included in REDCap. Residents were able to provide responses in binary (Yes/No), or Likert scales of 3, 5 or 6 categories, depending on the question. The physical and mental health scores range from 0 to 100, with a higher score indicating a better quality of life. However, the SF-12 questionnaire has been developed to compare participant’s scores with an average score of 50 and an SD of 10 in the adult US population.^27 28^

### Data management and statistical analysis

**Figure 1:**
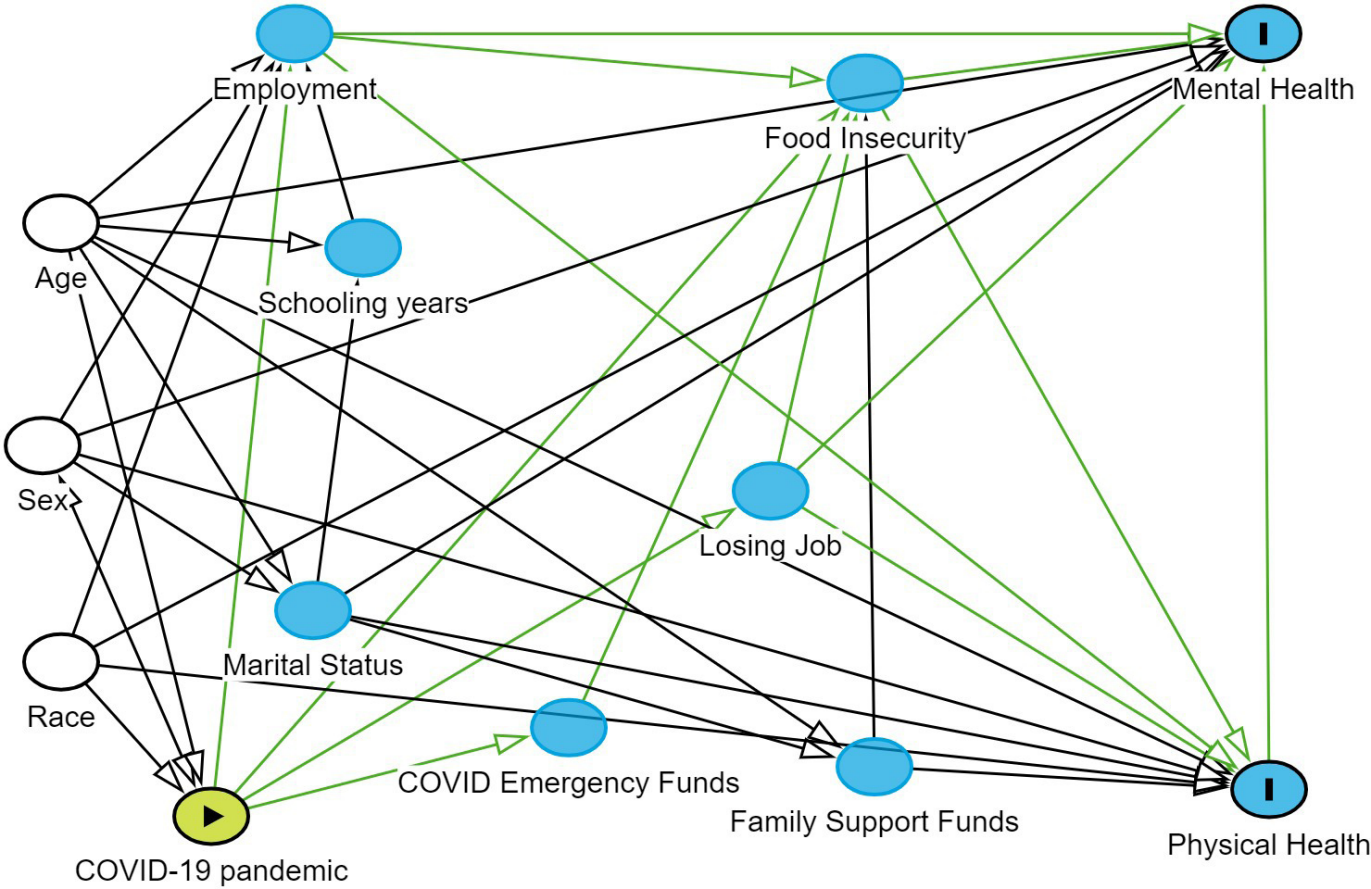
A Directed acyclic graph (DAG) showing the pathways linking the explanatory and dependent variables.

Briefly, we extracted data on the following variables: age, gender, marital status, educational status, race, food insecurity status, access to government’s financial aid (Bolsa Familia), access to COVID-19 emergency fund (Auxilio Emergencia), consequences of the pandemic, as perceived by respondents, on general well-being, mental health and the community, employment status before, during and after the lockdown and the 12 indicators of quality of life. Physical and mental quality of life represent dependent variables, while other variables were treated as independent, potentially explanatory variables. We created a directed acyclic graph (DAG) to show the pathways linking the explanatory and dependent variables based on the underlying hypothesis that the pandemic impacted on quality of life through socioeconomic indicators such as employment and food insecurity (figure 1). Our DAG identified 18 potential causal pathways, suggesting adjustment for age, sex and race as confounders, while employment, food insecurity and losing of job during the pandemic as mediators. No collider was identified in the DAG. All variables were subsequently imported for analysis in RStudio. Foremost, the dependent variables’ responses were processed separately for physical health and mental health questions according to suggested guidelines.^27 28^ This process involved applying regression coefficients from the general US population to weight individual responses, which were further aggregated into raw scores. To align the raw scores with the average values of the general US population, a regression intercept was added for standardisation. The dataset generated from this study can be found in online supplemental file 1 comprising the independent variables and the two dependent continuous variables (physical health score and mental health score).^29^

**Figure 2:**
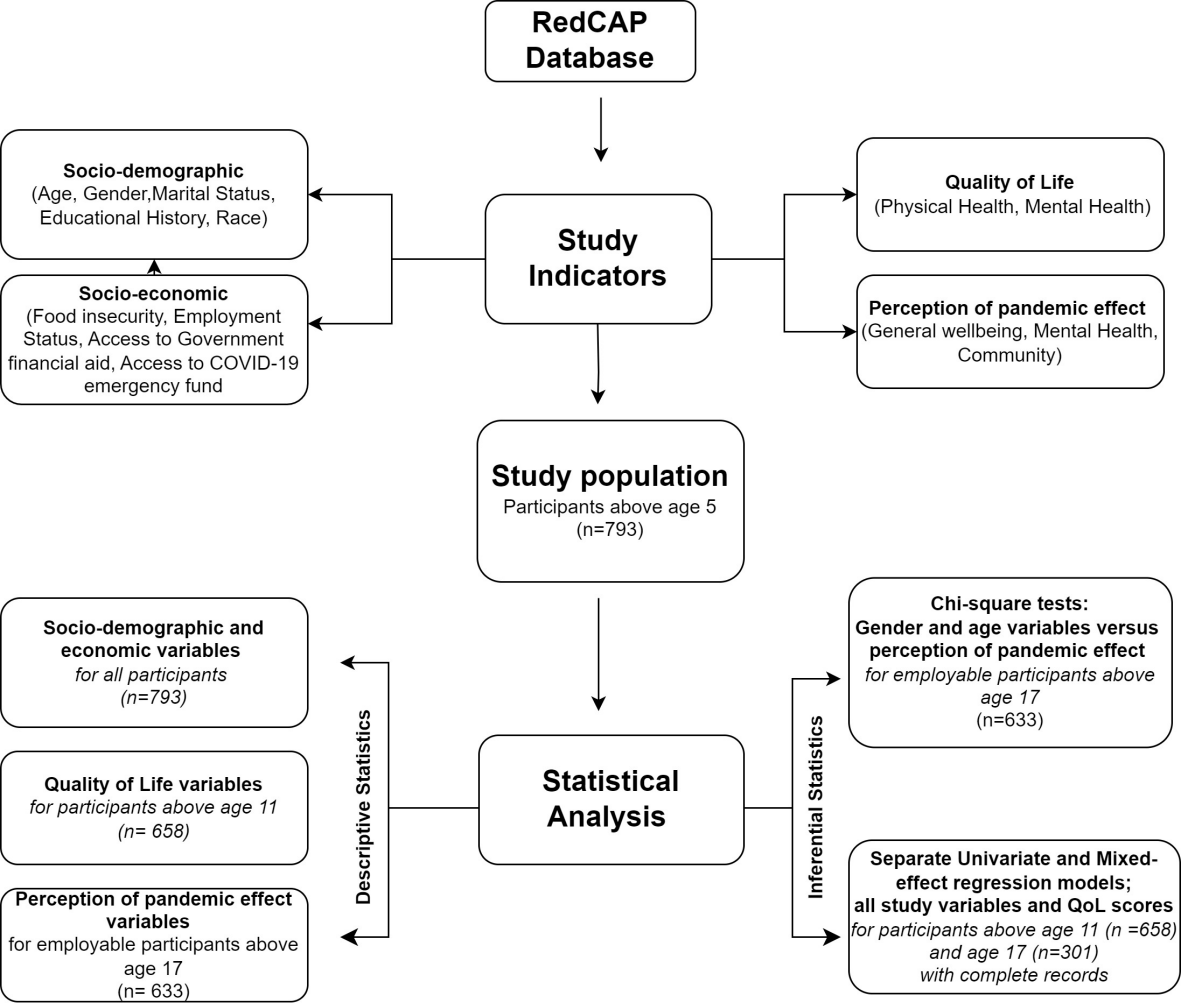
Flow chart of data management and analysis. QoL, quality of life.

For the statistical analysis, we employed a systematic approach (figure 2). We cross-tabulated the mean and SD scores of the dependent variables (quality of life scores) with independent variables. Associations were tested using t-tests or analyses of variance as appropriate. Furthermore, we filtered our datasets into two categories based on the age of participants (>11 and >17 years) to capture associations between the pandemic and variations in employment status. We created four employment categories among participants with age above 17 years: became employed during the pandemic, lost employment during the pandemic, remained employed all through the pandemic and remained unemployed all through the pandemic. First, we performed univariate linear model analyses to investigate associations between the pandemic and changes in employment categories and the HRQOL scores of participants. Following this, we performed separate mixed-effects regression models for participants aged >11 and >17 years to identify factors associated with physical and mental health scores. Variables included in the multivariate analyses were those with p<0.2 in separate analyses of models with a single explanatory variable. Variables included in the multivariable model were chosen following a stepwise selection method with households set as a random effect. Confounders and multicollinearity were evaluated using the Variance Inflation Factor (VIF) function. Regression estimates and 95% CIs were calculated and adjusted by the random effect. Significant association was established when p<0.05. In addition, using descriptive and χ^2^ tests, we explored the perceptions of participants regarding the consequences of the pandemic on mental health, and a variety of aspects of their general well-being including family and social relationships, education, finances, employment, hunger, violence, security and access to healthcare. All analyses were performed in R v.4.3.2 and the analysis code can be found in online supplemental file 2.^29^

### Patient and public involvement

This study specifically engages asymptomatic community residents rather than patients. Participants actively contributed to the research process, starting from the conceptualisation, site selection and community entrance before the initiation of study procedures. Considering our team’s established history in the study communities, the objectives were shaped around the priorities of infectious diseases like leptospirosis, focusing on the dam that traverses the community. The community, represented by established local structures and an identified representative, actively participated in decision-making processes. They also accompanied all field teams during household surveys for sample and data collection. Dissemination meetings were also conducted in the communities, involving community leaders and members, to share results and gather relevant feedback.

## Results

### Demographic characteristics of study participants

A total of 793 residents above age 5 participated in this study. Most of them were female (467, 58.9%), 357 between ages 18 and 45 years (45%), 350 self-declared black (44.2%) and 366 mixed race (46.3%). By educational profile, 337 of them (42.6%) had attained more than nine schooling years, and about one-third (284, 35.9%) had only attained the first 6 years of schooling. About one-third of those aged 18 and above were married (217, 34.3%). By employment status, almost half of those aged 18 and above were employed before the pandemic (313, 49.7%), but 38.8% of these lost their employment during the pandemic; 51.9% were employed at the time of the assessment. The prevalence of food insecurity was 69.6%, and only 27.1% and 54.7% of the participants had access to government aids (Bolsa Familia) and the COVID-19 emergency fund (Auxilio Emergencia), respectively (table 1).

**Table 1:** Demography and health-related quality of life (HRQOL) scores of participants

			HRQOL scores for participants aged >11 (n=658)			
		Total recruited	Physical health score*		Mental health score*	
		N=793 (%)	Mean±SD*	P value	Mean±SD*	P value
Gender	Male	326 (41.1)	48.6±9.7	0.021	49.2±8.2	<0.001
	Female	467 (58.9)	46.7±10.8		45.4±10.2	
Age	5–17	160 (20.2)	53.2±4.3	<0.001	46.2±9.8	0.084
	18–45	357 (45)	49.9±8.6		46.3±9.9	
	46–64	187 (23.6)	43.5±11.9		47.3±9.5	
	>64	89 (11.2)	41.9±11.7		49.1±7.9	
Race	White	60 (7.6)	46.1±9.6	0.432	49.7±6.5	0.072
	Black	350 (44.2)	47.9±10.6		46.8±9.9	
	Mixed race	366 (46.3)	47.0±10.4		46.9±9.6	
	Others	15 (1.9)	50.2±9.3		42.4±9.3	
Schooling years	None	34 (4.3)	38.4±12	0.000	51.6±7.3	0.063
	1–6	284 (35.9)	43.8±11.8		47.0±8.9	
	7–9	136 (17.2)	49.5±9.5		46.4±10.1	
	>9	337 (42.6)	49.4±8.8		46.7±9.9	
Married†	Yes	217 (34.3)	45.6±11	0.024	48.2±8.9	0.016
	No	416 (65.7)	48.3±10		46.3±9.9	
Currently employed†	Yes	327 (51.9)	48.6±9.5	<0.001	47.6±9.7	0.081
	No	303 (48.1)	44.9±11.5		46.3±9.7	
Employed before the pandemic†	Yes	313 (49.7)	49.2±8.7	<0.001	47.7±9.6	0.09
	No	317 (50.3)	44.6±11.9		46.3±9.6	
Lost employment during the pandemic‡	Yes	121 (38.8)	48.5±9.4	0.177	45.7±10.6	0.004
	No	191 (61.2)	49.8±8.1		48.9±8.7	
Stayed without food in the house during the pandemic§	Yes	535 (69.6)	46.7±10.6	0.043	46.7±9.6	0.527
	No	234 (30.4)	48.5±10.2		47.2±9.8	
Received family support fund§	Yes	215 (27.1)	47.4±10.0	0.943	45.0±9.8	0.003
	No	578 (72.9)	47.4±10.6		47.6±9.5	
Received COVID emergency fund§	Yes	434 (54.7)	47.2±10.4	0.468	46.0±9.6	0.007
	No	359 (45.3)	47.7±10.5		48.0±9.5	

* HRQOL analysis and scores reported for participants above age 11 (n=658).

† Data reported only for participants above age 17 (n=633).

‡ Data reported for participants who were employed before the pandemic (n=313).

§ Responses obtained from head of households and applied to all other household members.

Ntotal number of persons recruited

### HRQOL scores among study participants

Overall, participants had a mean score of 47.0±10.0 for both physical and mental health. The scores were significantly lower among females compared with males for physical health (46.7±10.8 vs 48.6±9.7, p=0.021) and mental health (45.4±10.2 vs 49.2±8.2, p<0.001). The scores for physical health declined as the age of participants increased (p<0.001), unlike for mental health where the scores tended to increase with participant age (p=0.084). However, the scores for physical health increased as participants attained more schooling years (p<0.001), unlike for mental health where the scores decreased (p=0.063). Participants who were married had reduced physical health scores (45.6±11 vs 48.3±10, p<0.05), but increased mental health scores (48.2±8.9 vs 46.3±9.9, p<0.05) when compared with those who were not married. Also, those who were employed had better physical (48.6±9.5 vs 44.9±11.5, p<0.001) and mental health scores (47.6±9.7 vs 46.3±9.7, p=0.081) when compared with those who were unemployed (table 1).

### Relationship between demography, socioeconomic and HRQOL scores among study participants during the pandemic

Table 2 shows the selected models following separate mixed-effects regression models on the relationship between sociodemographic and economic variables and HRQOL in all those >11 years of age, and in only those >17 years of age, where employment history could be included. In the full dataset, females had physical health scores lower than those of males (β: −2.44, 95% CI −3.94 to −0.94, p=0.002). Participants between ages 18 and 45 had physical health scores lower than younger participants (β: −4.09, 95% CI −6.96 to −1.22, p=0.005), and those who were above age 46 had the lowest physical health scores (β: −9.11, 95% CI −12.14 to −6.07, p<0.001). Participants who had food insecurity concerns also had physical health scores which were lower than those without such concern (β: −1.65, 95% CI −3.22 to −0.01, p=0.005). These patterns of association were also observed in the model with employable participants only (>17 years of age: model 2). Further, in this model, participants with higher schooling years had increased physical health scores (p<0.001). These patterns were especially pronounced among the older, employable participants (table 2).

**Table 2:** Relationship between demography, socioeconomic and health-related quality of life (HRQOL) scores among study participants during the pandemic

Model parameters	Model 1*		Model 2†	
	β (95% CI)	P value	β (95% CI)	P value
**Variables**				
**Physicalhealth**				
Intercept	46.10 (44.5, 54.6)	<0.001	52.11 (47.6, 56.6)	<0.001
Sex				
Male	Ref		Ref	
Female	−2.44 (−3.94, −0.94)	0.002	−2.29 (−4.17, −0.41)	0.01
Age				
12–17	Ref		NI	
18–45	−4.09 (−6.96, −1.22)	0.005	Ref	
46–64	−9.11 (−12.14, −6.07)	<0.001	−2.68 (−4.88, −0.41)	0.018
>64	−9.17 (−12.81, 5.52)	<0.001	−0.04 (−4.51, 4.42)	0.984
Schooling years				
None	Ref		Ref	
1–6	4.57 (0.63, 8.51)	0.024	12.3 (2.74, 21.9)	0.012
7–9	6.60 (2.27, 10.94)	0.003	14.8 (5.06, 24.6)	0.003
>9	7.38 (3.22, 11.55)	<0.001	15.5 (5.9, 25.1)	0.002
Food restrictions during pandemic				
No	Ref		NS	NS
Yes	−1.65 (−3.22, −0.01)	0.005	NS	NS
**Mentalhealth**				
Intercept	46.89 (45.18, 48.61)	<0.001	50.77 (48.74, 52.75)	<0.001
Sex				
Male	Ref	–		
Female	−2.12 (−3.72, −0.52)	0.009	−1.46 (−4.07, 1.29)	0.232
Married				
Yes	Ref		NS	NS
No	2.49 (0.78, 4.21)	0.004	NS	NS
Lost employment during the pandemic				
No	NS	NS	Ref	
Yes	NS	NS	−2.32 (−4.89, 0.24)	0.07

* Model using datasets with 658 complete cases for participants above age 11 to capture associations among those who are under employable age.

† Model using datasets with 301 complete cases for participants above age 17 to capture associations among those who are employable.

NI, not included in the model; NS, not selected in the final model; β, regression coefficient

Furthermore, females had mental health scores lower than those of males (β: −2.12, 95% CI −3.72 to −0.52, p=0.009); those who were not married had mental health scores higher than those who were married (β: 2.49, 95% CI 0.78 to 4.21, p=0.004); and among those who were employable, those who lost their job during the pandemic had mental scores which were lower compared with those who never did (β: −2.32, 95% CI −4.89 to 0.24, p=0.07) (table 3).

**Table 3:** Relationship between employment status and health-related quality of life (HRQOL) scores among study participants during the pandemic

	n (%)	Physical health scores			Mental health scores		
		Mean±SD	β (95% CI)	P value	Mean±SD	β (95% CI)	P value
Became employed*	84 (13.4)	45.6±11.7	Ref		45.3±10.6	Ref	
Lost employment†	121 (19.2)	48.5±9.4	2.64 (−0.25 to 5.53)	0.07	45.7±10	0.41 (−2.23 to 3.05)	0.76
Remained employed‡	191 (30.4)	49.8±8.1	4.02 (1.34 to 6.69)	<0.001	48.9±8.7	3.08 (0.60 to 5.56)	0.014
Remained unemployed§	233 (37.0)	44.1±11.9	−1.75 (−4.35 to 0.85)	0.187	46.7±9.2	1.06 (−1.32 to 3.46)	0.382
P value		0.000			0.005		

* : uUnemployed before the pandemic, but is currently employed;.

† : eEmployed before the pandemic, but lost the employment during the pandemic;.

‡ : eEmployed before the pandemic and still currently employed;.

§ : uUnemployed before the pandemic and still currently unemployed.

βregression coefficient

### Relationship between employment status and HRQOL scores among study participants during the pandemic

Table 3 shows the HRQOL of study participants based on employment categories. Of the 629 participants who were above 18 and eligible to work, 233 (37.0%) remained unemployed through the pandemic, that is, were unemployed before the pandemic and during the assessment. Also, 191 (30.4%) of the participants remained employed, but 121 (19.2%) who were employed before the pandemic lost their job during the pandemic. Only 84 (13.4%) of the participants became employed during the pandemic. There was a significant and positive relationship between remaining employed and physical health scores (β: 4.02, 95% CI 1.34 to 6.69, p<0.001). The score was lower among those who remained unemployed (44.1), and those who remained employed had the highest score (49.8). Furthermore, there was also a significant and positive relationship between remaining employed and mental health scores (β: 3.08, 95% CI 0.60 to 5.56, p<0.001). The score was lowest among participants who became employed (45.3) or lost their employment during the pandemic (45.7), while those who remained employed had the highest scores (48.9).

### Association between gender, age and difficulties experienced by participants during the pandemic

Tables4  5 show the perception of participants regarding the main challenges posed by the pandemic to themselves and to the community. Almost half of the employable participants reported that the pandemic impacted on their family relationships (47.4%), while 232 (36.7%) reported an impact on social relationships, 215 (34.0%) on work, 160 (25.3%) on health and 159 (25.1%) on children’s education (table 4). The gender and age-based association analysis shows that females perceived greater difficulties with health (p=0.009), children’s education (p<0.0001), family (p=0.06) and social relationship (p=0.06). Participants between ages 46 and 64 years perceived greater difficulty with work (p<0.0001) and children’s education (p<0.0001). Furthermore, participants reported that some of the major challenges COVID-19 brought to their community were felt on finance (390, 61.6%), employment (341, 53.9%), hunger (240, 37.9%), violence (211, 33.3%), security (206, 32.5%), access to health (209, 33%)and transportation (171, 27.0%)(table 5). The gender and age-based association analysis shows that females perceived greater difficulties with access to health (p=0.0031), while participants between ages 46 and 64 years perceived greater difficulties with finances (p=0.047) and employment (p=0.031).

**Table 4:** Association between gender, age and difficulties experienced by participants during the pandemic

Difficulties	N=633	Gender			Age group (in years)			
		Male(n=247)	Female(n=386)	P value	18–45(n=89)	46–64(n=357)	>64(n=187)	P value
Family relationships	300 (47.4)	105 (42.5)	195 (50.5)	0.060	40 (44.9)	172 (48.2)	88 (47.1)	0.856
Social relationships	232 (36.7)	79 (31.9)	153 (39.6)	0.060	26 (29.2)	134 (37.5)	72 (38.5)	0.284
Children’s education	159 (25.1)	40 (16.2)	119 (30.8)	0.000	16 (17.9)	110 (30.8)	33 (17.6)	0.000
Work/job	215 (34.0)	92 (37.2)	123 (31.9)	0.190	11 (12.4)	145 (40.6)	59 (31.6)	0.000
Health	160 (25.3)	48 (19.4)	112 (29.0)	0.009	24 (26.9)	81 (22.7)	55 (29.4)	0.213
Others	129 (20.4)	53 (21.5)	76 (19.7)	0.662	29 (32.6)	61 (17.1)	39 (20.9)	0.005
P value	<0.001	<0.011			<0.001			

**Table 5:** Association between gender, age and challenges brought by the pandemic to the community

Challenges	N=633	Gender			Age group (in years)			
		Male(n=247)	Female(n=386)	P value	18–45(n=89)	46–64(n=357)	>64(n=187)	P value
Finances	390 (61.6)	153 (61.9)	237 (61.4)	0.957	46 (51.7)	233 (65.3)	111 (59.4)	0.047
Employment	341 (53.9)	128 (51.8)	213 (55.2)	0.456	40 (44.9)	208 (58.3)	93 (49.7)	0.032
Hunger		84 (34.0)	156 (40.4)	0.124	31 (34.8)	145 (40.6)	64 (34.3)	0.279
Violence	211 (33.3)	74 (29.9)	137 (35.5)	0.176	31 (34.8)	118 (40.6)	62 (33.2)	0.949
Security	206 (32.5)	75 (30.4)	131 (33.9)	0.396	24 (26.9)	124 (34.73)	58 (31.0)	0.326
Access to health	209 (33.0)	64 (25.9)	145 (37.6)	0.003	31 (34.8)	125 (35.0)	53 (28.3)	0.269
Taking care of children	130 (20.5)	45 (18.2)	85 (22.0)	0.292	15 (16.9)	84 (23.5)	31 (16.6)	0.106
Lack of transportation	171 (27.0)	58 (23.5)	113 (29.3)	0.131	23 (25.8)	100 (28.0)	48 (25.7)	0.813
P value	0.001	0.569			0.874			

## Discussion

The global response to COVID-19 has primarily focused on containment measures,^13 17^ but the impact on the physical and mental well-being of individuals has received limited attention.^30^ This is particularly pronounced in urban slums, necessitating a greater emphasis on understanding the repercussions and addressing the challenges to improve quality of life and prepare for future pandemics. To address this issue, we used the SF-12 questionnaire to assess the HRQOL scores. This tool provides a comprehensive understanding of an individual’s overall well-being, considering both the physical and psychological aspects of their life.

The results of our study reveal that the overall HRQOL score among the slum population is 47, which is below the reference mean value of 50. While there is a scarcity of studies specifically investigating HRQOL in slum communities, our findings indicate lower scores compared with the most recent prepandemic studies conducted in Brazil. These previous studies reported mean scores ranging from 49 to 50 for physical health and 52 to 53 for mental health.^31 32^ The results of our study therefore suggest a reduction in HRQOL during the pandemic, particularly among females, elderly individuals, those who were married and those who were unemployed—differentials which align with previous studies from Brazil^32 33^ and elsewhere.^34^,^36^ We acknowledge, though, that the lack of any direct comparison between the same community before and during/after the pandemic precludes any direct attribution of cause.

More specific to the pandemic, however, there was a strong association between loss of employment during the pandemic and lower HRQOL scores. Furthermore, the perceived harmful consequences of the pandemic were focused on health, children’s education, and family and social relationship, especially in women, and on work and children’s education among those older than 46; and on access to health in the community especially among women, on problems with finances and employment especially among those older than 46, and on hunger, violence and security across the community.

Possible explanations for the disparity between the sexes include the influence of cultural norms and social factors on women,^37^ or increased responsibilities for household chores which could have negative impact of physical health, and indirectly reduce income opportunities, hence limiting their access to healthcare.^36 38^ Moreover, males tend to have better access to informal employment sectors or labour-intensive work, leading to their greater representation in the workforce within slum areas, whereas women often find themselves in poorly paid, temporary and exploitative jobs, facing restricted mobility, threats to their security and personal safety and exclusion from decision-making.^39^ These disparities may be reinforced by patriarchal power structures, which allow men to hold positions of authority and control over community affairs and decision-making processes, contributing to the gender imbalance in slum settings. Milner *et al*^40^ demonstrated reduced mental health in gender-dominated environments compared with neutral ones. There are also emerging thoughts that biological contributors such as hormonal changes during puberty, before menstruation or after pregnancy may trigger depression.^41^

Physical health scores of participants declined as age increased, while those of mental health increased with age. This is in line with the well-known phenomenon of age-related decline in skeletal muscle mass and maximal muscle force,^42 43^ and other studies noting that ageing people tend to exhibit better coping strategies, improved wisdom, emotional balancing and problem-solving skills.^44^,^48^ We further observed a positive association between physical health and years of schooling, while mental health showed a negative association. One plausible explanation for this finding is that higher levels of education provide individuals with access to better employment opportunities that require less physical activity, higher income and better working conditions. However, the decrease in mental health scores with increasing schooling years may be attributed to the challenges and increased responsibilities associated with demanding job roles. Another potential explanation could be that unemployment and limited job opportunities contribute to decline in mental health scores, as participants who experienced loss of employment during the pandemic reported lower mental health scores compared with those who remained employed. The detrimental consequence of the pandemic on quality of life may therefore be exacerbated among individuals with higher levels of educational attainment that are unemployed.

We also observed a very high prevalence of food insecurity. Prior to the pandemic, there has been an ongoing advocacy regarding the prevailing hunger epidemic in Brazil, with high malnutrition-induced mortality among children under 5, especially in the north-eastern region where this study was conducted.^21^ Addressing food insecurity is a crucial public health issue that requires attention. The Brazilian government has thus instituted the ‘Bolsa Familia’ support programme to provide monthly stipends for needy families in slums, and the ‘Auxilio Emergencia’ as additional stipends to support families during the COVID-19 pandemic. Nevertheless, our findings indicate that only one-third of the participants currently benefit from the Bolsa Familia programme. Additionally, the COVID-19 emergency fund, which was specifically established during the pandemic, reached only 55% of the participants. It is noteworthy that those who receive these funds are among the most vulnerable in the community, as they have lower HRQOL scores.

Overall, the COVID-19 pandemic has been accompanied by a worsening of the vulnerability of populations, particularly those already vulnerable, with participants reporting severe concerns regarding family and social relationships,^49^ finance, employment, hunger and violence. Hence, it is crucial to prioritise the creation of economic opportunities for marginalised populations residing in these slums. This includes contingency initiatives such as job creation, as well as the continued sustenance and expansion of existing assistance programmes. By doing so, we can effectively support the disadvantaged communities as we navigate the transition into the postpandemic era.

This study has several notable limitations. First, it adopts a cross-sectional design, which hinders the establishment of causality due to data being collected at a single time point. Second, the use of the SF-12 questionnaire, which was not specifically developed for the Brazilian population, or the unique conditions of slum areas, poses another limitation. The questionnaire may not fully capture the nuanced challenges faced by individuals living in Brazilian slums, potentially leading to incomplete or inaccurate assessments of their HRQOL. Additionally, the study was conducted during the post-lockdown era of the pandemic, raising the possibility of recall bias among participants concerning events that occurred during that period. This bias may affect the accuracy and reliability of the data collected. Despite these limitations, the study does offer valuable insights into the current quality of life situation in the slums of the study area. It serves as a foundation for further research and potential interventions aimed at addressing the unique challenges faced by this vulnerable population.

## Conclusion

In this study, we found lower HRQOL among adults, females, the unemployed and those with lower school attainment. In addition, women and individuals in older age groups reported experiencing COVID-impacted mental challenges more frequently than others. These findings highlight the need to prioritise creation of economic opportunities and expansion of existing assistance programmes for marginalised populations residing in these slums. This study therefore provides the baseline information on which further investments can be built.

## supplementary material

10.1136/bmjph-2023-000572 online supplemental file 1

10.1136/bmjph-2023-000572 online supplemental file 2

## Data Availability

Data are available in a public, open access repository.
